# Short-Term Classification Learning Promotes Rapid Global Improvements of Information Processing in Human Brain Functional Connectome

**DOI:** 10.3389/fnhum.2019.00462

**Published:** 2020-01-14

**Authors:** Antonio G. Zippo, Isabella Castiglioni, Jianyi Lin, Virginia M. Borsa, Maurizio Valente, Gabriele E. M. Biella

**Affiliations:** ^1^Institute of Molecular Bioimaging and Physiology, Consiglio Nazionale delle Ricerche, Milan, Italy; ^2^Department of Mathematics, Khalifa University, Abu Dhabi, United Arab Emirates; ^3^Department of Human and Social Sciences, University of Bergamo, Bergamo, Italy

**Keywords:** short-term memory, functional magnetic resonance imaging, functional connectivity, complex network analysis, information processing

## Abstract

Classification learning is a preeminent human ability within the animal kingdom but the key mechanisms of brain networks regulating learning remain mostly elusive. Recent neuroimaging advancements have depicted human brain as a complex graph machinery where brain regions are nodes and coherent activities among them represent the functional connections. While long-term motor memories have been found to alter functional connectivity in the resting human brain, a graph topological investigation of the short-time effects of learning are still not widely investigated. For instance, classification learning is known to orchestrate rapid modulation of diverse memory systems like short-term and visual working memories but how the brain functional connectome accommodates such modulations is unclear. We used publicly available repositories (openfmri.org) selecting three experiments, two focused on short-term classification learning along two consecutive runs where learning was promoted by trial-by-trial feedback errors, while a further experiment was used as supplementary control. We analyzed the functional connectivity extracted from BOLD fMRI signals, and estimated the graph information processing in the cerebral networks. The information processing capability, characterized by complex network statistics, significantly improved over runs, together with the subject classification accuracy. Instead, *null*-learning experiments, where feedbacks came with poor consistency, did not provoke any significant change in the functional connectivity over runs. We propose that learning induces fast modifications in the overall brain network dynamics, definitely ameliorating the short-term potential of the brain to process and integrate information, a dynamic consistently orchestrated by modulations of the functional connections among specific brain regions.

## Introduction

Learning sensory inputs is a crucial property for humans and animals in order to adapt their behaviors in relation to the external environment variability and survival (Tetzlaff et al., 2012). In many cases, these conditions demand for fast learnings which occur in short temporal intervals (i.e., from seconds to minutes). One specific type of learning (*classification learning*) requires classifications of objects into categories, an objective typically achievable by providing an adequate number of correctly labeled examples. For instance, an ornithology untrained subject can quickly learn to discriminate robins from songbirds after an opportune instruction with examples of both species.

In the human brain, classification learning essentially involves two memory systems: the visual working memory and the visual short-term memory (Knowlton et al., 1994; Foerde et al., 2007). The brain functional correlates of these systems have been broadly identified by functional magnetic resonance imaging (fMRI) as a distributed network in many cortical regions (Bettencourt and Xu, 2016) such as the prefrontal cortex, the middle temporal gyrus, the posterior parietal areas, and the occipital regions. Also, several subcortical regions like the hippocampus (Aron et al., 2004), the amygdala and the striatum seem involved, too (Harrison and Tong, 2009).

From a complex network perspective, the human brain regions have massive mutual dependencies, combined in a spatial organization arrangement known as the *modules-and-hubs* architecture (Bullmore and Sporns, 2009, 2012; van den Heuvel and Sporns, 2013), which promotes a wide variety of coherent subnetwork dynamics and tasks serving resting-state (Greicius et al., 2003), attention (Dosenbach et al., 2007; Sheremata et al., 2018), salience (Fox and Raichle, 2007), sensorimotor inputs and outputs (Bressler and Menon, 2010), language (Friederici and Gierhan, 2013) and other functions (Achard et al., 2006). However, the precise spatial and temporal superposition of each subsystem during cognitive tasks still remains unclear, possibly because of their reciprocal interconnections (Karahanoğlu and Van De Ville, 2015) which generate blurring intersections of their dynamic profile. Specifically, although particularly fine, statistical techniques such as the Independent Component Analysis (ICA) (Calhoun et al., 2001; Stone et al., 2002) may identify coherent activities in the spatial and temporal domains, the formal model relies on substantial assumptions as, for example, the non-Gaussianity of data, a limitation likely to be changed by a pure complex network approach which models the brain as a sole whole unit.

Therefore, in this work, we propose a functional connectome investigation of global large-scale neurophysiological bases of the visual working and short-term memory dynamics elicited by a classification learning task. In classification learning, human subjects have to learn, in few minutes, the association of some visual stimuli to specific choices (e.g., keystroke between two buttons). User choices are driven by visual feedbacks which lead the learning process. If feedbacks are consistently provided over time (*deterministically*), participants eventually learn the associations between visual stimuli and the correct responses, while, if feedbacks are administered by chance (*probabilistically*), participants do not learn any associations. We used fMRI data from publicly available repositories related to two similar classification learning experiments performed by Poldrack et al. (2001) (Aron et al., 2006) on 30 healthy subjects recruited. In a first part, the participants learned the proper stationary associations by visual error feedbacks in two consecutive sets of trials (Run 1 and 2) where the classification accuracy increased over runs (Poldrack et al., 2001; Aron et al., 2006). Subsequently, participants were challenged in another couple of run sets with non-stationary visual feedbacks, which disrupted the already formed memories and prevented the formation of new associations. As control condition, we used a third dataset where a cognitive task not activating short-term memory systems (one-back working memory task) was similarly executed along two runs. Examiners acquired the Blood-Oxygen-Level Dependent (BOLD) signal from fMRI together with a preliminary structural MRI of each subject. Functional connectomes of subjects were extracted for each run after the AFNI (Analysis of Functional NeuroImages) preprocessing pipeline (Cox, 2012) and embedded in two different atlases (Harvard-Oxford FSL (Rademacher et al., 1992; Fischl et al., 2004) and Brainnetome (Fan et al., 2016) to reduce effects of the choice of the anatomical parcellation. On the extracted graphs, we applied a common set of complex network statistics widely used in the brain functional connectomes (i.e., node degree, global and local efficiency, clustering coefficient and the average shortest path length) to investigate the information processing dynamics of the brain large-scale networks. Specifically, the aforementioned measures complementarily estimated the extent of functional segregation and functional integration (Tononi et al., 1994), two crucial statistics highlighting the information processing capability of complex brain networks.

The results showed a consistent and significant increment of the information processing efficiency in terms of functional segregation and integration in the second runs as compared to the first ones. This suggest that distributed and ample functional connectivity modifications emerge also in fast short-term learning, enabling faster information integration in classification learning tasks. Of note, these effects were mediated by coherent co-activation or deactivation of specific brain regions mainly from temporal, fusiform insular gyri and parietal lobe. No evident session effects emerged from the third dataset of one-back memory task.

## Materials and Methods

### Subject Data

Data were retrieved by the OpenfMRI project [openfmri.org now converged into the openneuro.org portal, number “ds002” (Poldrack et al., 2001) and “ds052” (Aron et al., 2006)] managed by the Poldrack Lab and the Center for Reproducible Neuroscience at Stanford University (United States). The third dataset was the “ds107” were uploaded by Duncan et al. (2009). The database and its contents are made available under the Public Domain Dedication and License v1.0 (PDL)^1^. The ds002 dataset was populated by 17 healthy right-handed participants (female = 10, age = 23.3 ± 2.8). The ds052 dataset contained 13 healthy subjects (female = 7, age = 22.8 ± 3.2). The ds107 dataset contained 49 healthy monolingual English speakers. For each participant, both fMRI acquisitions (repetition time, TR, of 2.0 s in ds002 and ds052 and of 3.0 s in ds107, echo time, TE, of 4 ms in ds002 and ds052 and of 50 ms in ds107) and structural MRIs were included. All details about MRI and BOLD acquisitions can be found in the related works (Poldrack et al., 2001; Aron et al., 2006; Duncan et al., 2009). Classification accuracy of experiments ds002 and ds052 has been computed by the metadata contained in the ds002 and ds052 datasets.

### Deterministic and Probabilistic Classification Tasks

**FIGURE 1:**
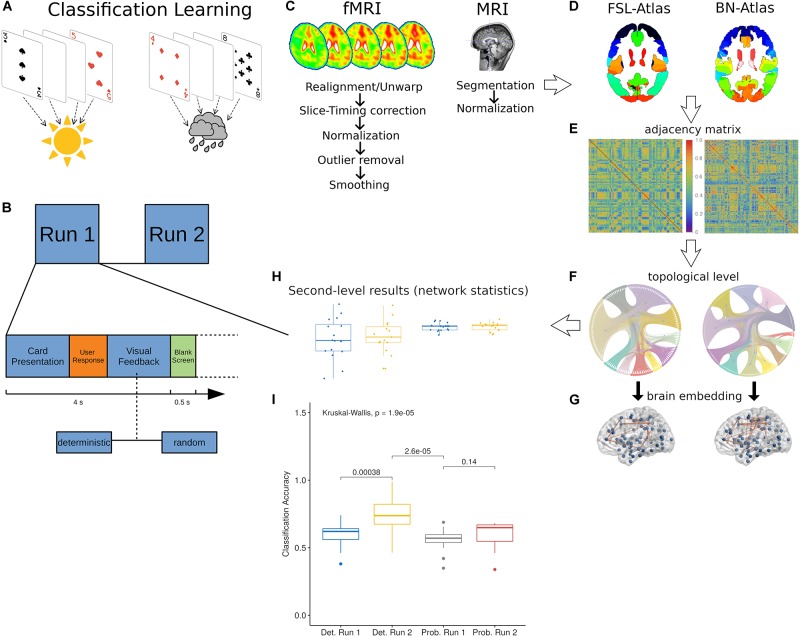
The experimental and computational frameworks. **(A)** Healthy participants performed a weather-prediction task through the association of card types to a binary weather output (sunny/rainy). **(B)** Two stages of trials were presented sequentially to subjects where each trial was composed by four sections: a first phase characterized by the visual presentation of the card, a second stage wherein the user makes the choice (sun/rain), a third phase with the visual feedback (correct/wrong) and a short final rest phase with a blank screen. Depending on the task type, the feedbacks could be assigned deterministically or probabilistically. **(C)** The AFNI preprocessing pipeline used for the structural MRI and the BOLD signals. **(D)** Axial view samples of the two atlases used to parcellate the fMRI volumes: the FSL and the Brainnetome (BN). **(E–G)** Examples of, respectively, adjacency matrices **(E)**, their related topological **(F)** and MNI space embeddings **(G)**. **(H)** Exemplary collections of complex network statistics plotted in Box–Whisker (1st, 25th, 50th, and 99th percentiles) with scattering points as measure of dispersion. **(I)** The classification accuracy reported by the original works (Poldrack et al., 2001; Aron et al., 2006) shows that probabilistic feedbacks did not evoke any consistent association learning.

The objective of a classification learning experiment is to promote the learning of a set of associations between visual stimuli and specific user responses. Learning is driven by visual feedbacks which lead the participant to choose responses that give back “correct” feedbacks. When feedbacks are consistently provided over the experimental session (i.e., with a deterministic assignment), participants eventually learn the associations between visual stimuli and correct response while, if feedbacks are administered by chance (i.e., by a probabilistic assignment), participants do not learn the arbitrary associations. During the fMRI scans in the ds002 and ds052 experiments, the subjects had to perform two different classification learning tasks along two consecutive runs in a “weather prediction” setup (Figure 1A). Participants have to learn associations between cards (four in ds002, one to three in ds052) and a binary output, visually represented (as feedback) by a sun or a rainy cloud, after their responses. Learning occurs trial-by-trial while the visual feedback errors (correct/incorrect) drive subjects towards the correct card-weather associations. According to the metadata in the dataset, although these consistently derives from the materials presented in Poldrack et al. (2001), Aron et al. (2006), in the first series of two consecutive runs, trials were characterized by deterministic associations between cards and weathers. In the second series, instead, the associations were assigned probabilistically. In each run there were 80 trials in ds002 (∼5 min of total duration) and 48 in ds052 (∼3 min of total duration).

### One-Back Working Memory Task

In the ds107 experiment, the participants (*N* = 49) observe a sequence of objects and have to press a specific key on a keyboard whether the current object was identical to the previous one, during MRI scanning. Visual stimuli belonged to four categories (Duncan et al., 2009): written words, pictures of common objects, scrambled pictures and consonant letter strings. Stimuli were presented in a sequence of four blocks. Each block consisted of 16 trials from a single category. Objects appeared on a screen for 350 ms each. A trial began with a 650 ms fixation cross, for a total of 1 s per trial.

### Signal Processing

Data were preprocessed and analyzed using the following MATLAB toolbox: SPM12 (Friston et al., 2007), CONN (Whitfield-Gabrieli and Nieto-Castanon, 2012) and BCT (Rubinov and Sporns, 2010). Prior to analyses, all images underwent preprocessing steps according to the AFNI pipeline (Cox, 2012) in the following order: realign and unwarp of functional slices, centering of functional slices, slice-timing correction of functional volumes, outlier detection in functional volumes, direct segmentation and normalization in Montreal Neurological Institute (MNI) space of the functional volumes, centering of the structural slices, segmentation and normalization in MNI space of the structural volumes and smoothing of the functional volumes. We used the default parameters (functional outlier detection = 97th percentiles, global-signal *z*-value threshold = 5, subject-motion mm threshold = 0.9, structural target resolution = 1 mm, functional target resolution = 2 mm, smoothing kernel FWHM = 8 mm) suggested within the CONN framework for all processing steps (Whitfield-Gabrieli and Nieto-Castanon, 2012). In addition, to avoid errors derived from the choice of the atlas, we used two different atlases: the FSL (Rademacher et al., 1992; Fischl et al., 2004) and the Brainnetome (Fan et al., 2016) (BN). From the FSL atlas we removed ROIs related to the Vermis and the Cerebellum thus obtaining 106 ROIs, while the BN atlas did not contain cerebellar regions and consists of 246 ROIs.

### Functional Connectivity Estimation

After BOLD signal preprocessing, data underwent a denoising step through a band-pass filter in the frequency of [0.008, 0.09] Hz and a despiking procedure to furtherly remove motion artifacts after the ArtiFact detection tool (ART)-based scrubbing^2^ (Power et al., 2012; Van Dijk et al., 2012; Jo et al., 2013). Voxelwise time series were transformed into region of interest (ROIs) series by averaging the signal over all ROI voxels. Two parcellation atlas were used in this study: the FSL (Fischl et al., 2004) and the Brainnetome (Fan et al., 2016). In the subsequent first-level analysis, we computed the ROI-to-ROI connectomes (by means of the CONN toolbox) represented by adjacency matrices obtained through a bivariate analysis of the Pearson correlation coefficient between all ROI couples transformed with the Fischer *z*-transformation (setting to 0 those with a False Discovery Rate, FDR (Benjamini and Hochberg, 1995), corrected *p*-value larger than 0.05). Formally, given *R*_*i*_(*t*) the *ith* (of *n* distinct) ROI BOLD signal measured at the *tth* scan, results of the averaging of all voxels within the *ith* ROI (centered for zero mean, i.e., by subtracting the estimated mean value), *r* (Pearson’s correlation coefficient) and *Z* are defined as follow:

$$r\,\left(i,j\right)=\frac{\sum_{t=1}^{T}R_{i}\,\left(t\right)\,R_{j}\,\left(t\right)}{\sqrt{\sum_{t=1}^{T}R_{i}\,{\left(t\right)}^{2}\,\sum_{t=1}^{T}R_{j}\,{\left(t\right)}^{2}}},$$

*Z*(*i*,*j*)=*tanh*^−1^⁡*r*(*i*,*j*), with *i*,*j* = 1,2,⋯,*n* and *T* is the total number of fMRI scans.

For each subject and condition, *Z* is the adjacency matrix of the resulting graph *G* = ⟨*V*,*E*⟩ where *V* = {*v*_*i*_:*i* = 1, 2,⋯,*n*} is the set of all ROIs and *E*={*e*_*i*,*j*_|∀*v*_*i*_,*v*_*j*_ ∈ *V*} is the set of all edges, the functional connectome of interest which comprised all *i*,*j* ROI’s couples. Summarily, a node *v*_*i*_ of the graph *G* denotes the *ith* ROI, while an edge *e*_*i,j*_ connecting nodes *v*_*i*_ and *v*_*j*_ is computed using the Z transform of the Pearson correlation coefficient between the ROIs *R*_*i*_(*t*) and *R*_*j*_(*t*) such that *e*_*i,j*_. All graphs were maintained in their weighted form.

We analyzed the functional connectivity graphs (i.e., the *Z* matrices) with a set of common network statistics (node degree, global and local efficiency, clustering coefficient and the average shortest path length) by avoiding thresholding techniques which provoke loss of information and makes analyses more complicated because of the introduction of the threshold parameter (Rubinov and Sporns, 2011). For both atlases, we selected only forebrain regions (Tables 1, 2 for details) by excluding the cerebellum because its ubiquitous role (E et al., 2014) in high cognition is still debated (Yu et al., 2015; Gelal et al., 2016).

**TABLE 1:** Region of interest labels and coordinates in MNI space of the FSL Atlas (CONN default).

**Label ID**	**Coordinates (*X*, *Y*, *Z*)**	**Name (Abbreviation)**
1	26, 52, 8	Frontal Pole Right (FP r)
2	−25, 53, 8	Frontal Pole Left (FP l)
3	37, 3, 0	Insular Cortex Right (IC r)
4	−36, 1, 0	Insular Cortex Left (IC l)
5	15, 18, 57	Superior Frontal Gyrus Right (SFG r)
6	−14, 19, 56	Superior Frontal Gyrus Left (SFG l)
7	39, 19, 43	Middle Frontal Gyrus Right (MidFG r)
8	−38, 18, 42	Middle Frontal Gyrus Left (MidFG l)
9	52, 28, 8	Inferior Frontal Gyrus, pars triangularis Right (IFG tri r)
10	−50, 28, 9	Inferior Frontal Gyrus, pars triangularis Left (IFG tri l)
11	52, 15, 16	Inferior Frontal Gyrus, pars opercularis Right (IFG oper r)
12	−51, 15, 15	Inferior Frontal Gyrus, pars opercularis Left (IFG oper l)
13	35, −11, 50	Precentral Gyrus Right (PreCG r)
14	−34, −12, 49	Precentral Gyrus Left (PreCG l)
15	41, 13, −30	Temporal Pole Right (TP r)
16	−40, 11, −30	Temporal Pole Left (TP l)
17	58, −1, −10	Superior Temporal Gyrus, anterior division Right (aSTG r)
18	−56, −4, −8	Superior Temporal Gyrus, anterior division Left (aSTG l)
19	61, −24, 2	Superior Temporal Gyrus, posterior division Right (pSTG r)
20	−62, −29, 4	Superior Temporal Gyrus, posterior division Left (pSTG l)
21	58, −2, −25	Middle Temporal Gyrus, anterior division Right (aMTG r)
22	−57, −4, −22	Middle Temporal Gyrus, anterior division Left (aMTG l)
23	61, −23, −12	Middle Temporal Gyrus, posterior division Right (pMTG r)
24	−61, −27, −11	Middle Temporal Gyrus, posterior division Left (pMTG l)
25	58, −49, 2	Middle Temporal Gyrus, temporooccipital part Right (toMTG r)
26	−58, −53, 1	Middle Temporal Gyrus, temporooccipital part Left (toMTG l)
27	46, −2, −41	Inferior Temporal Gyrus, anterior division Right (aITG r)
28	−48, −5, −39	Inferior Temporal Gyrus, anterior division Left (aITG l)
29	53, −23, −28	Inferior Temporal Gyrus, posterior division Right (pITG r)
30	−53, −28, −26	Inferior Temporal Gyrus, posterior division Left (pITG l)
31	54, 50, −17	Inferior Temporal Gyrus, temporooccipital part Right (toITG r)
32	−52, −53, −17	Inferior Temporal Gyrus, temporooccipital part Left (toITG l)
33	38, −26, 53	Postcentral Gyrus Right (PostCG r)
34	−38, −28, 52	Postcentral Gyrus Left (PostCG l)
35	29, −48, 59	Superior Parietal Lobule Right (SPL r)
36	−29, −49, 57	Superior Parietal Lobule Left (SPL l)
37	58, −27, 38	Supramarginal Gyrus, anterior division Right (aSMG r)
38	−57, −33, 37	Supramarginal Gyrus, anterior division Left (aSMG l)
39	55, −40, 34	Supramarginal Gyrus, posterior division Right (pSMG r)
40	−55, −46, 33	Supramarginal Gyrus, posterior division Left (pSMG l)
41	52, −52, 32	Angular Gyrus Right (AG r)
42	−50, −56, 30	Angular Gyrus Left (AG l)
43	33, −71, 39	Lateral Occipital Cortex, superior division Right (sLOC r)
44	−32, −73, 38	Lateral Occipital Cortex, superior division Left (sLOC l)
45	46, −74, −2	Lateral Occipital Cortex, inferior division Right (iLOC r)
46	−45, −76, −2	Lateral Occipital Cortex, inferior division Left (iLOC l)
47	12, −74, 8	Intracalcarine Cortex Right (ICC r)
48	−10, −75, 8	Intracalcarine Cortex Left (ICC l)
49	0, 43, −19	Frontal Medial Cortex (MedFC)
50	6, −3, 58	Supplementary Motor Cortex Right (SMA r)
51	−5, −3, 56	Supplementary Motor Cortex Left (SMA l)
52	0, 21, −15	Subcallosal Cortex (SubCalC)
53	7, 37, 23	Paracingulate Gyrus Right (PaCiG r)
54	−6, 37, 21	Paracingulate Gyrus Left (PaCiG l)
55	1, 18, 24	Cingulate Gyrus, anterior division (AC)
56	1, −37, 30	Cingulate Gyrus, posterior division (PC)
57	1, −59, 38	Precuneous Cortex (Precuneous)
58	9, −79, 28	Cuneal Cortex Right (Cuneal r)
59	−8, −80, 27	Cuneal Cortex Left (Cuneal l)
60	29, 23, −16	Frontal Orbital Cortex Right (FOrb r)
61	−30, 24, −17	Frontal Orbital Cortex Left (FOrb l)
62	22, −8, −30	Parahippocampal Gyrus, anterior division Right (aPaHC r)
63	−22, −9, −30	Parahippocampal Gyrus, anterior division Left (aPaHC l)
64	23, −31, −17	Parahippocampal Gyrus, posterior division Right (pPaHC r)
65	−22, −32, −17	Parahippocampal Gyrus, posterior division Left (pPaHC l)
66	14, −63, −5	Lingual Gyrus Right (LG r)
67	−12, −66, −5	Lingual Gyrus Left (LG l)
68	31, −3, −42	Temporal Fusiform Cortex, anterior division Right (aTFusC r)
69	−32, −4, −42	Temporal Fusiform Cortex, anterior division Left (aTFusC l)
70	36, −24, −28	Temporal Fusiform Cortex, posterior division Right (pTFusC r)
71	−36, −30, −25	Temporal Fusiform Cortex, posterior division Left (pTFusC l)
72	35, −50, −17	Temporal Occipital Fusiform Cortex Right (TOFusC r)
73	−33, −54, −16	Temporal Occipital Fusiform Cortex Left (TOFusC l)
74	27, −75, −12	Occipital Fusiform Gyrus Right (OFusG r)
75	−27, −77, −14	Occipital Fusiform Gyrus Left (OFusG l)
76	41, 19, 5	Frontal Operculum Cortex Right (FO r)
77	−40, 18, 5	Frontal Operculum Cortex Left (FO l)
78	49, −6, 11	Central Operculum Cortex Right (CO r)
79	−48, −9, 12	Central Operculum Cortex Left (CO l)
80	49, −28, 22	Parietal Operculum Cortex Right (PO r)
81	−48, −32, 20	Parietal Operculum Cortex Left (PO l)
82	48, −4, −7	Planum Polare Right (PP r)
83	−47, −6, −7	Planum Polare Left (PP l)
84	46, −17, 7	Heschl’s Gyrus Right (HG r)
85	−45, −20, 7	Heschl’s Gyrus Left (HG l)
86	55, −25, 12	Planum Temporale Right (PT r)
87	−53, −30, 11	Planum Temporale Left (PT l)
88	8, −74, 14	Supracalcarine Cortex Right (SCC r)
89	−8, −73, 15	Supracalcarine Cortex Left (SCC l)
90	18, −95, 8	Occipital Pole Right (OP r)
91	−17, −97, 7	Occipital Pole Left (OP l)
92	11, −18, 7	Thalamus Right (Thalamus r)
93	−10, −19, 6	Thalamus Left (Thalamus l)
94	13, 10, 10	Caudate Right (Caudate r)
95	−13, 9, 10	Caudate Left (Caudate l)
96	25, 2, 0	Putamen Right (Putamen r)
97	−25, 0, 0	Putamen Left (Putamen l)
98	20, −4, −1	Palladium Right (Palladium r)
99	−19, −5, −1	Palladium Left (Palladium l)
100	26, −21, −14	Hippocampus Right (Hippocampus r)
101	−25, −23, −14	Hippocampus Left (Hippocampus l)
102	23, −4, −18	Amygdala Right (Amygdala r)
103	−23, −5, −18	Amygdala Left (Amygdala l)
104	9, 12, −7	Accubens Right (Accubens r)
105	−9, 11, −7	Accubens Left (Accubens l)
106	0, −30, −35	Brainstem (Brainstem)

**TABLE 2:** ROI labels and coordinates in MNI space of the Brainnectome Atlas (BN).

**Gyrus**	**Brodmann’s location**	**Label ID.L**	**Label ID.R**	**MNI.L**	**MNI.R**
Superior Frontal Gyrus	A8m, medial area 8	1	2	−5, 15, 54	7, 16, 54
	A8dl, dorsolateral area 8	3	4	−18, 24, 53	22, 26, 51
	A9l, lateral area 9	5	6	−11, 49, 40	13, 48, 40
	A6dl, dorsolateral area 6	7	8	−18, −1, 65	20, 4, 64
	A6m, medial area 6	9	10	−6, −5, 58	7, −4, 60
	A9m, medial area 9	11	12	−5, 36, 38	6, 38, 35
	A10m, medial area 10	13	14	−8, 56, 15	8, 58, 13
Middle Frontal Gyrus	A9/46d, dorsal area 9/46	15	16	−27, 43, 31	30, 37, 36
	IFJ, inferior frontal junction	17	18	−42, 13, 36	42, 11, 39
	A46, area 46	19	20	−28, 56, 12	28, 55, 17
	A9/46v, ventral area 9/46	21	22	−41, 41, 16	42, 44, 14
	A8vl, ventrolateral area 8	23	24	−33, 23, 45	42, 27, 39
	A6vl, ventrolateral area 6	25	26	−32, 4, 55	34, 8, 54
	A10l, lateral area 10	27	28	−26, 60, −6	25, 61, −4
Inferior Frontal Gyrus	A44d, dorsal area 44	29	30	−46, 13, 24	45, 16, 25
	IFS, inferior frontal sulcus	31	32	−47, 32, 14	48, 35, 13
	A45c, caudal area 45	33	34	−53, 23, 11	54, 24, 12
	A45r, rostral area 45	35	36	−49, 36, −3	51, 36, −1
	A44op, opercular area 44	37	38	−39, 23, 4	42, 22, 3
	A44v, ventral area 44	39	40	−52, 13, 6	54, 14, 11
Orbital Gyrus	A14m, medial area 14	41	42	−7, 54, −7	6, 47, −7
	A12/47o, orbital area 47	43	44	−36, 33, −16	40, 39, −14
	A11l, lateral area 11	45	46	−23, 38, −18	23, 36, −18
	A11m, medial area 11	47	48	−6, 52, −19	6, 57, −16
	A13, area 13	49	50	−10, 18, −19	9, 20, −19
	A12/47l, lateral area 12/47	51	52	−41, 32, −9	42, 31, −9
Precentral Gyrus	A4hf, area 4(head and face region)	53	54	−49, −8, 39	55, −2, 33
	A6cdl, caudal dorsolateral area 6	55	56	−32, −9, 58	33, −7, 57
	A4ul, area 4(upper limb region)	57	58	−26, −25, 63	34, −19, 59
	A4t, area 4(trunk region)	59	60	−13, −20, 73	15, −22, 71
	A4tl, area 4(tongue and larynx region)	61	62	−52, 0, 8	54, 4, 9
	A6cvl, caudal ventrolateral area 6	63	64	−49, 5, 30	51, 7, 30
Paracentral Lobule	A1/2/3ll, area1/2/3 (lower limb region)	65	66	−8, −38, 58	10, −34, 54
	A4ll, area 4 (lower limb region)	67	68	−4, −23, 61	5, −21, 61
Superior Temporal Gyrus	A38m, medial area 38	69	70	−32, 14, −34	31, 15, −34
	A41/42, area 41/42	71	72	−54, −32, 12	54, −24, 11
	TE1.0 and TE1.2	73	74	−50, −11, 1	51, −4, −1
	A22c, caudal area 22	75	76	−62, −33, 7	66, −20, 6
	A38l, lateral area 38	77	78	−45, 11, −20	47, 12, −20
	A22r, rostral area 22	79	80	−55, −3, −10	56, −12, −5
Middle Temporal Gyrus	A21c, caudal area 21	81	82	−65, −30, −12	65, −29, −13
	A21r, rostral area 21	83	84	−53, 2, −30	51, 6, −32
	A37dl, dorsolateral area37	85	86	−59, −58, 4	60, −53, 3
	aSTS, anterior superior temporal sulcus	87	88	−58, −20, −9	58, −16, −10
Inferior Temporal Gyrus	A20iv, intermediate ventral area 20	89	90	−45, −26, −27	46, −14, −33
	A37elv, extreme lateroventral area 37	91	92	−51, −57, −15	53, −52, −18
	A20r, rostral area 20	93	94	−43, −2, −41	40, 0, −43
	A20il, intermediate lateral area 20	95	96	−56, −16, −28	55, −11, −32
	A37vl, ventrolateral area 37	97	98	−55, −60, −6	54, −57, −8
	A20cl, caudolateral of area 20	99	100	−59, −42, −16	61, −40, −17
	A20cv, caudoventral of area 20	101	102	−55, −31, −27	54, −31, −26
Fusiform Gyrus	A20rv, rostroventral area 20	103	104	−33, −16, −32	33, −15, −34
	A37mv, medioventral area 37	105	106	−31, −64, −14	31, −62, −14
	A37lv, lateroventral area 37	107	108	−42, −51, −17	43, −49, −19
Parahippocampal Gyrus	A35/36r, rostral area 35/36	109	110	−27, −7, −34	28, −8, −33
	A35/36c, caudal area 35/36	111	112	−25, −25, −26	26, −23, −27
	TL, area TL (lateral PPHC, posterior parahippocampal gyrus)	113	114	−28, −32, −18	30, −30, −18
	A28/34, area 28/34 (EC, entorhinal cortex)	115	116	−19, −12, −30	19, −10, −30
	TI, area TI(temporal agranular insular cortex)	117	118	−23, 2, −32	22, 1, −36
	TH, area TH (medial PPHC)	119	120	−17, −39, −10	19, −36, −11
posterior Superior Temporal Sulcus	rpSTS, rostroposterior superior temporal sulcus	121	122	−54, −40, 4	53, −37, 3
	TS, caudoposterior superior temporal sulcus	123	124	−52, −50, 11	57, −40, 12
Superior Parietal Lobule	A7r, rostral area 7	125	126	−16, −60, 63	19, −57, 65
	A7c, caudal area 7	127	128	−15, −71, 52	19, −69, 54
	A5l, lateral area 5	129	130	−33, −47, 50	35, −42, 54
	A7pc, postcentral area 7	131	132	−22, −47, 65	23, −43, 67
	A7ip, intraparietal area 7(hIP3)	133	134	−27, −59, 54	31, −54, 53
Inferior Parietal Lobule	A39c, caudal area 39(PGp)	135	136	−34, −80, 29	45, −71, 20
	A39rd, rostrodorsal area 39(Hip3)	137	138	−38, −61, 46	39, −65, 44
	A40rd, rostrodorsal area 40(PFt)	139	140	−51, −33, 42	47, −35, 45
	A40c, caudal area 40(PFm)	141	142	−56, −49, 38	57, −44, 38
	A39rv, rostroventral area 39(PGa)	143	144	−47, −65, 26	53, −54, 25
	A40rv, rostroventral area 40(PFop)	145	146	−53, −31, 23	55, −26, 26
Precuneus	A7m, medial area 7(PEp)	147	148	−5, −63, 51	6, −65, 51
	A5m, medial area 5(PEm)	149	150	−8, −47, 57	7, −47, 58
	dmPOS, dorsomedial parietooccipital sulcus(PEr)	151	152	−12, −67, 25	16, −64, 25
	A31, area 31 (Lc1)	153	154	−6, −55, 34	6, −54, 35
Postcentral Gyrus	A1/2/3ulhf, area 1/2/3(upper limb, head and face region)	155	156	−50, −16, 43	50, −14, 44
	A1/2/3tonIa, area 1/2/3(tongue and larynx region)	157	158	−56, −14, 16	56, −10, 15
	A2, area 2	159	160	−46, −30, 50	48, −24, 48
	A1/2/3tru, area1/2/3(trunk region)	161	162	−21, −35, 68	20, −33, 69
Insular Gyrus	G, hypergranular insula	163	164	−36, −20, 10	37, −18, 8
	vIa, ventral agranular insula	165	166	−32, 14, −13	33, 14, −13
	dIa, dorsal agranular insula	167	168	−34, 18, 1	36, 18, 1
	vId/vIg, ventral dysgranular and granular insula	169	170	−38, −4, −9	39, −2, −9
	dIg, dorsal granular insula	171	172	−38, −8, 8	39, −7, 8
	dId, dorsal dysgranular insula	173	174	−38, 5, 5	38, 5, 5
Cingulate Gyrus	A23d, dorsal area 23	175	176	−4, −39, 31	4, −37, 32
	A24rv, rostroventral area 24	177	178	−3, 8, 25	5, 22, 12
	A32p, pregenual area 32	179	180	−6, 34, 21	5, 28, 27
	A23v, ventral area 23	181	182	−8, −47, 10	9, −44, 11
	A24cd, caudodorsal area 24	183	184	−5, 7, 37	4, 6, 38
	A23c, caudal area 23	185	186	−7, −23, 41	6, −20, 40
	A32sg, subgenual area 32	187	188	−4, 39, −2	5, 41, 6
MedioVentral Occipital Cortex	cLinG, caudal lingual gyrus	189	190	−11, −82, −11	10, −85, −9
	rCunG, rostral cuneus gyrus	191	192	−5, −81, 10	7, −76, 11
	cCunG, caudal cuneus gyrus	193	194	−6, −94, 1	8, −90, 12
	rLinG, rostral lingual gyrus	195	196	−17, −60, −6	18, −60, −7
	vmPOS,ventromedial parietooccipital sulcus	197	198	−13, −68, 12	15, −63, 12
Lateral Occipital Cortex	mOccG, middle occipital gyrus	199	200	−31, −89, 11	34, −86, 11
	V5/MT+, area V5/MT+	201	202	−46, −74, 3	48, −70, −1
	OPC, occipital polar cortex	203	204	−18, −99, 2	22, −97, 4
	iOccG, inferior occipital gyrus	205	206	−30, −88, −12	32, −85, −12
	msOccG, medial superior occipital gyrus	207	208	−11, −88, 31	16, −85, 34
	lsOccG, lateral superior occipital gyrus	209	210	−22, −77, 36	29, −75, 36
Amygdala	mAmyg, medial amygdala	211	212	−19, −2, −20	19, −2, −19
	lAmyg, lateral amygdala	213	214	−27, −4, −20	28, −3, −20
Hippocampus	rHipp, rostral hippocampus	215	216	−22, −14, −19	22, −12, −20
	cHipp, caudal hippocampus	217	218	−28, −30, −10	29, −27, −10
Basal Ganglia	vCa, ventral caudate	219	220	−12, 14, 0	15, 14, −2
	GP, globus pallidus	221	222	−22, −2, 4	22, −2, 3
	NAC, nucleus accumbens	223	224	−17, 3, −9	15, 8, −9
	vmPu, ventromedial putamen	225	226	−23, 7, −4	22, 8, −1
	dCa, dorsal caudate	227	228	−14, 2, 16	14, 5, 14
	dlPu, dorsolateral putamen	229	230	−28, −5, 2	29, −3, 1
Thalamus	mPFtha, medial pre-frontal thalamus	231	232	−7, −12, 5	7, −11, 6
	mPMtha, pre−motor thalamus	233	234	−18, −13, 3	12, −14, 1
	Stha, sensory thalamus	235	236	−18, −23, 4	18, −22, 3
	rTtha, rostral temporal thalamus	237	238	−7, −14, 7	3, −13, 5
	PPtha, posterior parietal thalamus	239	240	−16, −24, 6	15, −25, 6
	Otha, occipital thalamus	241	242	−15, −28, 4	13, −27, 8
	cTtha, caudal temporal thalamus	243	244	−12, −22, 13	10, −14, 14
	lPFtha, lateral pre-frontal thalamus	245	246	−11, −14, 2	13, −16, 7

### Complex Network Statistics

For the analysis of the connectome graphs, we selected a set of common statistics from the Complex Network Theory able to estimate the network information processing extent. Table 3 shows measures (definition and interpretation) used in the present work. Analyses on the extracted functional brain networks were performed in Matlab by the Brain Connectivity Toolbox (BCT) (Rubinov and Sporns, 2010), by the Python *graph toolbox* (Peixoto, 2014c), and by other *ad hoc*-routines developed in our lab. Specifically, we used a complementary measure of information integration, called Compression Flow (CF), that we previously showed to effectively discriminate patients diagnosed with mild cognitive impairment from those with probable Alzheimer’s disease (Zippo et al., 2015). For a better numerical treatment of the results, the original fourth stage, consisting in a summation, was replaced by an average, as follows.

**TABLE 3:** The complex network statistics used in this work.

**Measure**	**Definition**	**Interpretation**
Node degree (also known as *node strength*)	$k_{i}=\sum_{j\in V}e_{i,j}$	The sum of weights connected to a given node *i*
Average Shortest path length	Given: $d_{i\,j}=\sum_{e_{f,g}\in r_{i↔j}}1/e_{f,g}$ where *r*_*i↔j*_ is the shortest path between *i* and *j*;$L=\frac{1}{n}\,\sum_{i\in V}\frac{\sum_{j\in V,j\neq i}d_{i\,j}}{n-1}$	The average edge weights encountered in the shortest path between node *i* and *j*
Local Efficiency (Latora and Marchiori, 2001)	$E_{l\,o\,c}=\frac{1}{n}\,\sum_{i=1}^{n}\frac{\sum_{j,z\in V,j\neq i}{\left(e_{i,j}\,e_{i,z}\,{\left[d_{i\,j}\,\left(N_{i}\right)\right]}^{-1}\right)}^{\frac{1}{3}}}{\left(k_{i}-1\right)\,k_{i}}$, where *d*_*ij*_(*N*_*i*_) is the length of the shortest path length between *i* and *j* that contains only nodes directly connected to *i*	Measure of local network segregation. Supplementary to the clustering coefficient
Global Efficiency (Latora and Marchiori, 2001)	$E=\frac{1}{n}\,\sum_{i=1}^{n}\frac{\sum_{j\in V,j\neq i}d_{i\,j}^{-1}}{n-1}$	Measure of network integration. The inverse of the average shortest path length that became meaningful in disconnected networks with infinite length paths
Clustering coefficient (Watts and Strogatz, 1998)	$C=\frac{1}{n}\,\sum_{i\in V}C_{i}=\frac{1}{n}\,\sum_{i\in V}\frac{2\,t_{i}}{k_{i}\,\left(k_{i}-1\right)}$, with $t_{i}=\frac{1}{2}\,\sum_{j,h\in V}\sqrt[{3}]{e_{i,j}\,e_{i,h}\,e_{j,h}}$	Measure of fine-grain network segregation. It counts the average weight of triangles *t* (3-node fully connected graphs) present in the network

*As assumption, *Z* is an adjacency matrix of the graph *G* = ⟨*V*,*E*⟩ with *V* = {*v*_*i*_:*i* = 1, 2,⋯,*n*} and *E*={*e*_*i*,*j*_|∀*v*_*i*_,*v*_*j*_ ∈ *V*}where the element *e*_*i,j*_ represents the connection weight between nodes *i* and *j*.*

Algorithm:

Inputs: *Z* is the adjacency matrix of the graph *G* = ⟨*V*,*E*⟩ with *V* = {*v*_*i*_:*i* = 1, 2,⋯,*n*} and *E*={*e*_*i*,*j*_|∀*v*_*i*_,*v*_*j*_ ∈ *V*}, the node betweenness centrality (BC) of *G*, and the edge betweenness centrality (EBC) of *G*;

Output: the extent of *CF* for the graph *G*.

Steps:

1. Set a pivot value ϑ in the BC distribution, usually a low percentile of the BC distribution (values from 5 to 20 do not affect results);
2. Establish which nodes have a BC lower than ϑ, thus obtaining the subset φ⊂*V* with |φ| = *k*of the putative most peripheral nodes of *G* (|■| the cardinality operator);
3. For *w* = 1,2,⋯,*k* compute and collect the random walks *r*_*w*_ from the periphery to the network center for each input load *w*; at each step the *w* activated nodes are randomly chosen from φ;
4. For *w* = 1,2,⋯,*k* estimate the compression ratio by computing (through the *c* function) and counting the number of connected components $\left|c\,\left(\hat{G}\right)\right|$ of the graph provisional $\hat{G}$ obtained by the collection of all edges encountered in all paths of *r*_*w*_; the compression ratio is set to $\rho_{w}=\frac{w}{n-\left|c\,\left(\hat{G}\right)\right|}$;
5. Average the obtained $C\,F=\frac{1}{k}\,\sum_{i=1}^{k}\rho_{i}$.

The algorithm is written in Matlab and the code are available upon request. The connected components of graphs are computed by a depth-first search algorithm.

From the graph toolbox we used an efficient routine to extract the hierarchical modularity from networks (Peixoto, 2014a,b, 2015a,b). The implemented algorithm (the stochastic block models) outperforms many other common modularity methods (Newman, 2006; Blondel et al., 2008) and it has been chosen for this reason.

### Statistical Tests

We performed statistical comparisons between the used complex network statistics within each experimental condition. Specifically, these included the type of feedback: probabilistic or deterministic, the chosen atlas: FSL or BN atlas and the dataset: ds002, ds052, or ds107.

For hypothesis testing, we made no assumption about the *a priori* data distribution, thus, we used non-parametric models. Pairwise comparisons were performed by the non-parametric Wilcoxon signed rank test with Bonferroni correction for multiple contrasts (by multiplying the *p*-values for the total number of hypotheses), while for multiple group comparisons we used the Kruskal–Wallis test with the False Discovery Rate (FDR) correction. The significance level was assumed as 0.05 in all hypothesis tests.

### Edge Filtering

To identify relevant edges (i.e., functional connections between ROIs) which supported the observed information processing enhancement from run 1 to run 2, we set up a statistical procedure that selected edges which significantly changed between runs. Edge weights were modeled by a linear model and fitted with univariate ANOVA criteria in the R language environment (R Core Team, 2019). Pairwise comparisons between runs were subsequently performed with the Tukey post-hoc test. Edges below the significance level (0.05) were furtherly filtered to select those with an absolute high magnitude. For this reason, we picked edges whose differences were either greater than the 95th percentile (namely, *positive differences*) or lesser than the 5th percentile (*negative differences*). Since, the difference weight distribution had about zero mean, the latter set grouped only edge with negative weights.

## Results

In the present work, we preprocessed fMRI volumes, from two classification learning experiments (Figures 1A,B), according to the AFNI pipeline (Figure 1C) and subsequently we extracted the ROI-to-ROI functional connectivity for each subject (Figures 1D–H) according to the two atlases templates (FSL and BN, coordinates in Tables 1, 2). The classification learning tasks were of two types: deterministic and probabilistic (Figures 1A,B). The former represented the actual classification learning assumed to occur in subjects (reported performances in Figure 1I), the second indicated the *null* hypothesis where learning was dampened through probabilistic feedbacks and thus memory associations were precluded. We considered a third experiment, as additional control, to evaluate the role of possible session-effects in a different cognitive task recruiting only visual working memory systems (visual one-back task). On the functional connectomes we computed a common set of complex network statistics (Table 3) to assess the network information processing capability between the first (run 1) and the second group (run 2) of trials revealing a significant increment of classification accuracy (Figure 1I). Eventually, we used a recently presented (Zippo et al., 2015) refined functional integration measure, of functional integration, the compression flow (CF), stochastically estimating the network capability to learn and predict external inputs.

**FIGURE 2:**
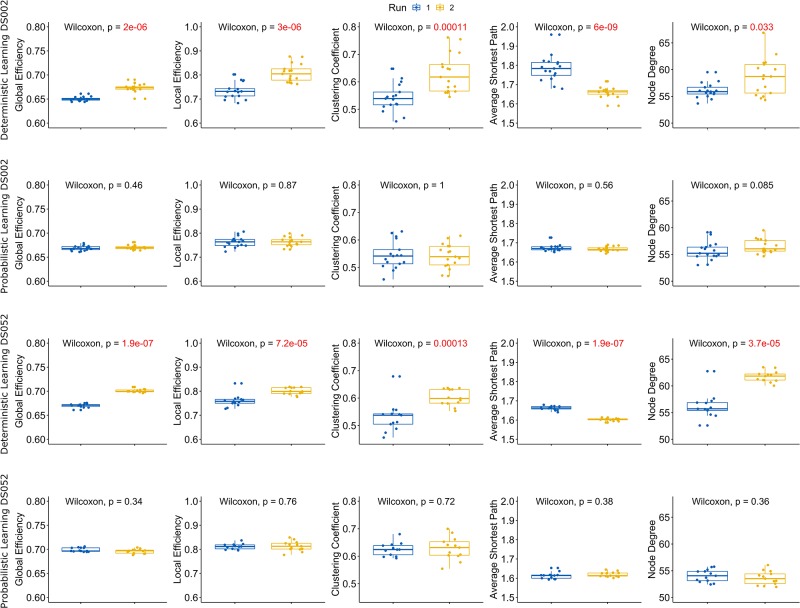
Complex Network statistics for FSL atlas. A complete overview of the complex network statistics (respectively, Global and Local Efficiency, Clustering Coefficient, Average Shortest Path Length and Node Degree see Table 3) computed on the functional connectomes for both datasets (ds002, ds052, rows 1–2 and 3–4, respectively) and both experimental conditions (deterministic/probabilistic) embedded in the FSL atlas. Plots reported the statistical significance according to the Wilcoxon signed rank test with Bonferroni correction. Boxplot colors indicate the run: blue for run 1 and yellow for run 2. Significant *p*-values (<0.05) are highlighted in red.

**FIGURE 3:**
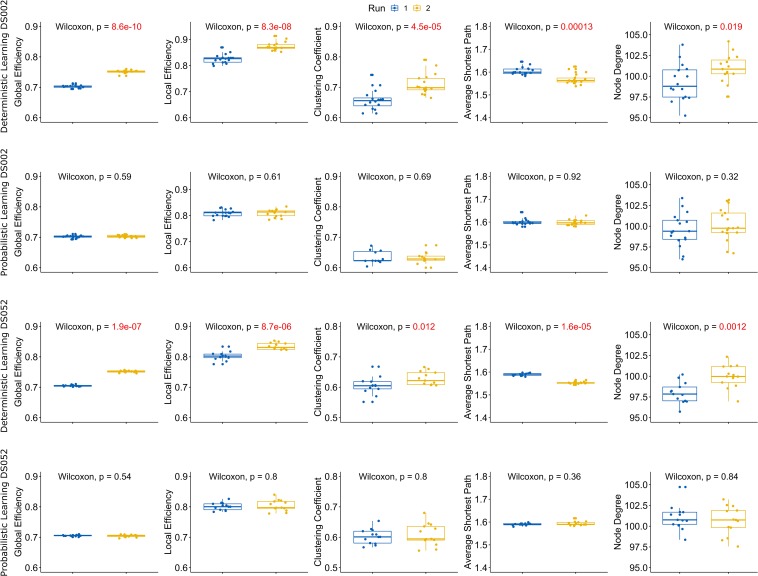
Complex Network statistics for BN atlas. A complete overview of the complex network statistics (respectively, Global and Local Efficiency, Clustering Coefficient, Average Shortest Path Length and Node Degree see Table 3) computed on the functional connectomes for both datasets (ds002, ds052, rows 1–2 and 3–4 respectively) and both experimental conditions (deterministic/probabilistic) embedded in the Brainnetome atlas. Plots reported the statistical significance according to the Wilcoxon signed rank test with Bonferroni correction. Boxplot colors indicate the run: blue for run 1 and yellow for run 2. Significant *p*-values (<0.05) are highlighted in red.

We found that, in deterministic sessions, the node degree distribution, the global and local efficiency, the clustering coefficient and the characteristic path length were all significantly different between runs. Specifically, the node degree distribution, the global and the local efficiency and the clustering coefficient were higher in run 2 while, conversely, the average shortest path length was smaller (results of Wilcoxon’s tests in Figures 2, 3, rows one and three). Moreover, analyses from both datasets (ds002, ds052) and both atlases (FLS, BN) generated congruent observations. In details, the node degree increment indicated that new functional connections were activated in the second run. The global and local efficiencies measured how proficiently the information was exchanged within respectively the entire graph and neighbor’s nodes. The observed efficiency dynamics suggested that, in run 2, information exchange was optimized thus minimizing the processing energetic expenditure (Bullmore and Sporns, 2012). The clustering coefficient changes, instead, demonstrated that the networks were more prone to segregate information in run 2 compared to run 1. Eventually, the characteristic path length decreasing in run 2 expressed a reduction in the average path length between node random couples. These outcomes indicated that the observed networks became more topologically efficient in run 2, and, therefore, according to complex network statistics, the brain networks became more effective in terms of information processing capabilities and more prone to integrate and segregate information. Conversely, in probabilistic trials (considered as control) we did not observe any significant modulations between runs (results of Wilcoxon’s tests in Figures 2, 3, rows two and four) in both datasets (ds002, ds052) and in both atlases (FLS, BN). Altogether, these results proposed a scenario where the brain functional connectome, when exerted by an input learning demand, alters its connections in order to optimize the information storage of the putative predictive associations (Tetzlaff et al., 2012).

**FIGURE 4:**
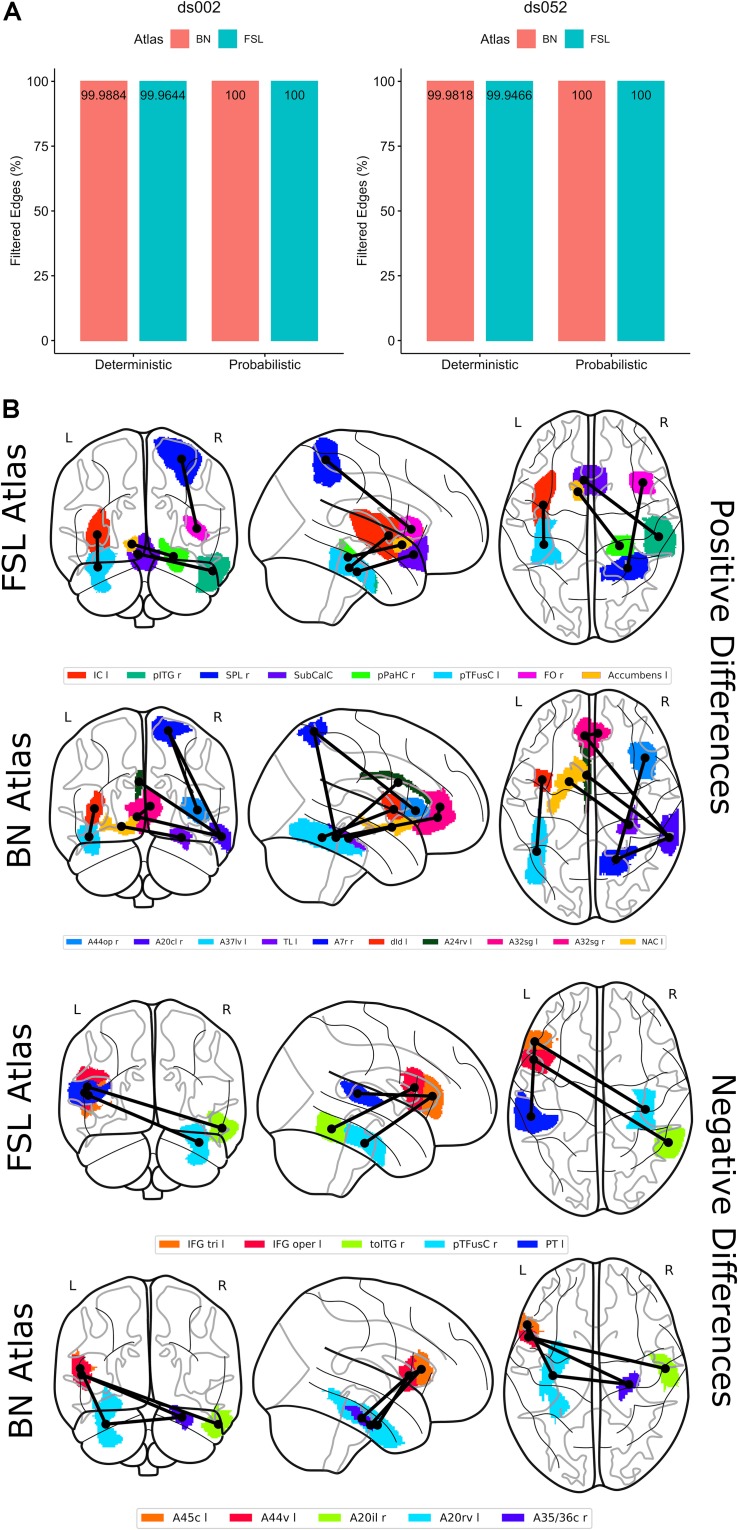
Selection of the most salient functional connections. An edge filtering procedure statistically selected the strongest (above the 95th percentile) and the weakest (below the 5th percentile) connections evoked in run 2. **(A)** Percentage of filtered edges in the diverse experimental conditions (deterministic/probabilistic), datasets (ds002, ds052) and atlases (FSL, BN). **(B)** The resulting edges with positive differences are shown in three different views (posterior, lateral, superior) in the first two rows with plot_glass_brain function of the nilearn python library. In the first row, results were extracted from the FSL atlas while in the second rows from the BN atlas. Connections are represented by black lines and the centroids of the regions of interest by small black circles. Similarly, the third and the fourth rows indicates the negative differences. ROI colors are chosen arbitrarily.

Subsequently, we wondered which putative functional connections shaped the observed network dynamics and, accordingly, we analyzed edge fluctuations between runs with statistical hypothesis tests (see section “Materials and Methods”). For more statistical robustness and consistent interpretation of the data, we combined sessions from both datasets ds002 and ds052. We found that only deterministic sessions identified statistically significant and remarkable connections (Figure 4A) and we divided these relevant edges into two sets: the first set containing edged tightly strengthened in run 2, the second containing the weakened edges in run 2. Within the FSL atlas (Figure 4B), we found four strengthened connections, namely the left insular cortex with the left temporal fusiform cortex, the left superior parietal lobe with the right frontal operculum, the right inferior temporal cortex with the subcallosal cortex and the left nucleus accumbens with the right parahippocampal gyrus. These results were coherent with results obtained with BN atlas which covered more than 80% of each correspondent brain regions. Instead, the weakened edges were those connecting the left inferior frontal gyrus with the right fusiform cortex, the right temporo-occipital inferior temporal cortex with the left inferior frontal gyrus and left planum temporale with the left inferior frontal gyrus. Again, these results were remarkably coherent with results obtained with BN atlas where brain regions between atlases were overlapped at least for 84%.

**FIGURE 5:**
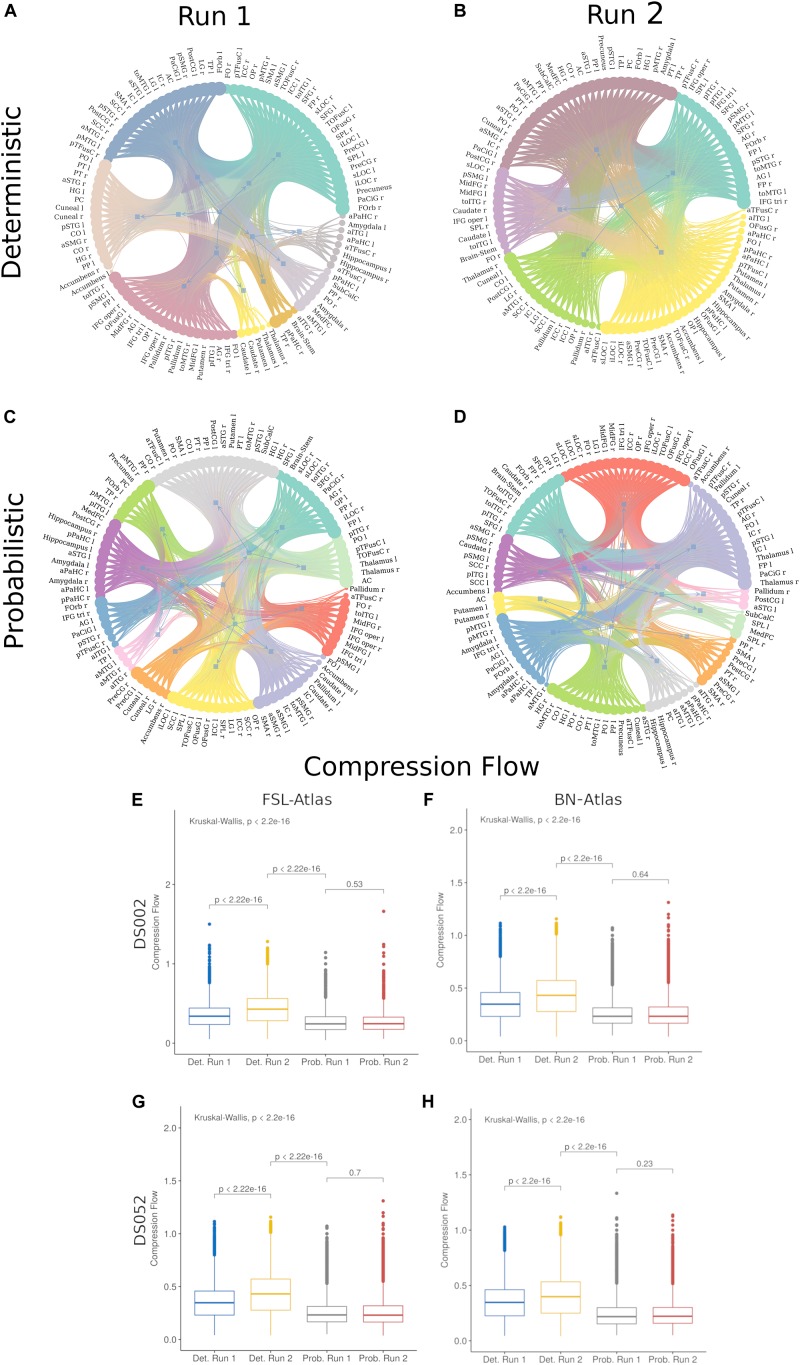
Hierarchical Modularity Structure and Compression Flow statistics. Hierarchical modularity analysis of the FSL grand average networks (run 1 vs. run 2, respectively, **A**, **B**) among subjects (*N* = 30) and experiments (ds002, ds052) for the deterministic condition. In run 2, the functional modules of the connectome collapse, as a sign of the arisen functional integration, into five communities with a singular hierarchical level, from the eight communities of run 1 arranged in two hierarchical levels (five modules in the second level). Oppositely, in probabilistic condition modular organization did not change **(C,D)**. Edge colors mark community membership and are arbitrarily chosen by the graph plotting routine. Analyses of the compression flow measure of brain graphs by using the FSL atlas **(E,G)** and the BN atlas **(F,H)** or the ds002 **(E,F)** and ds052 **(G,H)** experiments. Plots reported the statistical significance according to the Kruskal–Wallis non-parametric test with a False Discovery Rate (FDR) correction for group comparisons while, for pairwise comparisons, the Wilcoxon signed rank test significance with Bonferroni correction is reported. In **(E–H)**, Deterministic is referred with “Det.” and Probabilistic with “Prob.”. Boxplot colors (blue, yellow, gray, and red) denote the diverse conditions (respectively deterministic run 1, deterministic run 2, probabilistic run 1 and probabilistic run 2).

Looking for a further indication of the increment of information integration in run 2, we averaged all FSL connectomes for all participants of both experiments (ds002, ds052) and we analyzed the hierarchical modular organization of nodes in communities comparing deterministic and probabilistic conditions. We observed that the number of modules and hierarchical levels dropped from run 1 to run 2 indicating that network information processing took place in more integrated topological architectures (Figures 5A,B). Statistically, prior to averaging, we found 7.4 ± 1.9 (mean and standard deviation) modules in run 1 and 4.9 ± 0.7 in run 2 with a significant difference (*p* = 0.001, non-parametric Wilcoxon signed ranksum test). Vice versa, the number of modules did not decrease in the probabilistic conditions as a sign of a missed integrative merging among modules (Figures 5C,D, 8.1 ± 1.4 in run 1 vs. 7.8 ± 1.9 in run 2, *p* = 0.349, ranksum test). Ultimate, the CF estimations coherently confirmed the significant increment of the topological information integration of the connectomes (Figures 5E–H) in the second run of deterministic trials.

**FIGURE 6:**
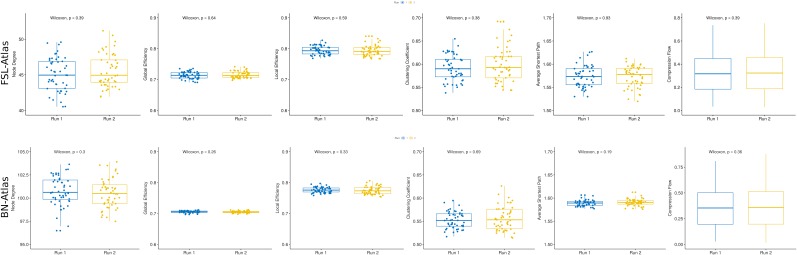
Complex Network Statistics in a non-classification learning cognitive task. The first row represents the collection of network statistics obtained by extracting the ROIs according to the FSL atlas, while in the second row the statistics are computed with the BN atlas. Altogether, the lack of statistically significant differences indicate that no session effect is present between run 1 and run 2. Boxplot colors indicate the run: blue for run 1 and yellow for run 2.

Eventually, to evaluate the possible role of the session-effect (run 1 vs. run 2), we decided to include a further dataset from the same repository (ds107) where participants performed a one-back working memory task. Results in Figure 6 did not show any significant differences (Wilcoxon’s test) confirming that observed results in the previous analyses were not merely an outcome of the comparison between runs.

Altogether these evidences suggest that critical topological modifications of the functional connectome allow large-scale architecture to accommodate the incoming cognitive demand achieving high efficiency with low energy expenditure.

## Discussion

In this work, we investigated the fast and transient topological dynamics of a short-term memory task in broad functional connectomes. We found a consistent enhancement of the functional integration and segregation during the trial-by-trial generation of the associative learning. Namely, connectomes became more efficient in information processing capability in diverse experimental conditions and analyses, a property absent in both sham and control experiments.

Therefore, as highlighted by our estimates and analyses, higher cognitive tasks involve global connectome adaptations rather than mere local topological modifications of few regions. This property implies a new assessment of general brain dynamics obliging to reconsider the conventional view of brain functional specialization as common refrain in studies correlating few but specific brain regions with distinct cognitive tasks. From a clinical perspective, this widespread interpretation takes its origins from old neurological judgements of past centuries, assuming a causality between anatomically observable lesions and specific disruptions of behavioral or cognitive functions. In contrast to such a perspective, the functional connectivity network of the human brain proposes a strong global interdependency among regions where alterations of single node dynamics may be echoed widespreadly over the entire network, significantly changing the brain network dynamics. The assumption of localized lesional models inevitably neglects the complex and diffuse damages upon the globally connected brain network, as well as the compensatory or repair mechanisms that, with diverse strength and at diverse time, may arise from the original alterations (Catricalà et al., 2015).

From a computational point of view, classification learning implies an information storage demand to be accomplished in short time intervals (from seconds to few minutes). Indeed, according to the Friston’s free-energy minimization principles (Friston, 2010), nervous systems work to minimize the discrepancy between external world information and the related internal brain representations in neuronal networks. This theoretical approach is, seemingly, time-independent and active at most different time-scales.

Furthermore, in our previous work we conjectured that compression flow is inversely related with the free-energy (Zippo et al., 2015), namely, when brain networks increase the extent of compression flow, the system free-energy decreases. Therefore, we could suggest that the new information needed by the classification learning task induces a bump of free-energy that, theoretically, is likely to be cut by means of topological modifications of the functional connectivity.

In the literature, as cited in the introduction of this work, original neurophysiological studies on non-human primates showed that visual working memory appeared solely related with the prefrontal cortex, the parietal cortex and some associative area in the occipital lobe. Subsequent studies further extended the list of the involved areas with the contribution of the premotor cortex, the intraparietal sulcus, the caudate, the hippocampus, the thalamus and several occipitotemporal regions (Doron et al., 2012). This progressive spatial extension of neuronal networks involved in visual tasks, primarily strengthens the global vs. local accounting of brain dynamics and, as a consequence leads to hypothesize a more extended design applicable to other systems and task conditions. From a more classical anatomical view it shows augmented functional connectivity within the rostro-caudal axis (Kuo et al., 2011) enriching dramatically the neuronal textures ignited by an external specific stimulus and functionally requires a widely distributed dense network for the active maintenance of a perceptual representation (Gazzaley et al., 2004).

By an edge-centric perspective, our results showed an enhancement of specific brain region connections. In particular the functional connection between the left insular and the left fusiform cortices appears in accordance with the putative roles of such districts implicated, respectively, in the consolidation of object recognition memory (Bermudez-Rattoni et al., 2005) and working memory tasks (Druzgal and D’Esposito, 2001; Postle et al., 2003; Hofer et al., 2007), two executive functions heavily recruited in classification learning assignments. Similarly, the strengthened connections between the right parahippocampal gyrus and the left nucleus accumbens are likely consonant with their putative roles such as, respectively, in short-term memory (Daselaar et al., 2001; Peters et al., 2009) and the visual memory consolidation (Setlow, 1997; Deadwyler et al., 2004). Again, the right frontal operculum and left superior parietal lobe, participating in task control (Higo et al., 2011), in episodic memory retrieval (Wagner et al., 2005) and in the maintaining of internal representation (Wolpert et al., 1998). Eventually, the right posterior inferior temporal cortex, a crucial region of the ventral stream visual processing directly involved in the object recognition (Gross, 1992; Nobre et al., 1994) with the subcallosal cortex responsible, instead, for the monitoring and the control of executive processes (Hebscher et al., 2016). In opposition, other functional connections were inhibited. Specifically, these connections encompass the inferior temporal and the fusiform cortices with the inferior frontal gyrus, usually recruited in response inhibition (Swick et al., 2008), in the selection among competing alternatives (Moss et al., 2005; Hirshorn and Thompson-Schill, 2006) and attentional control (Hampshire et al., 2010).

Past works with strong local-centric activated networks showed that there are two distinctly different stages in accessing information in short-term memory, a stage elicited in the classification learning, recruiting the inferior temporal regions with frontal- and posterior-parietal contributions, the medial temporal lobe and left mid-ventrolateral prefrontal cortex (Nee and Jonides, 2008). In contrast, van den Berg and coworkers stated that neural representation of visual short-term memory is continuous and variable rather than discrete and fixed, thus smoothing this modular interpretation (van den Berg et al., 2012). In addition, early evidences suggested that the cortico-limbic neurophysiological substrate of visual short-term memory changed globally, rather than with focal modifications, in healthy elderly subjects (Della-Maggiore et al., 2000). On this track, D’Esposito (2007) concluded (in a study on the visual working memory) that it is not localized to a single brain region but more likely it represents an emergent property of the functional interactions between the prefrontal cortex and the rest of the brain, a key step towards the shift of a local towards a global appraisal of brain functional domains. However, our results from a visual working memory task (one-back) seemed to encourage the idea that the observed global topological optimization causally emerged from the visual short-term rather than working memory completions.

Other studies on fast dynamics of the visual working memory suggested an involvement of several EEG frequency bands (α, β, γ) over large-scale densely connected cortical areas (frontal, parietal and occipital) for maintenance and coordination (Palva J. M. et al., 2010; Palva S. et al., 2010). These findings multiply and supply furtherly more complex pictures that succeed in a more temporally accurate technique (i.e., the EEG) which highlighted the dynamic complexity of the global brain involvement yet engaged in “simple” visual tasks. A novel work suggests, again, a cross-modal recruitment of sensory related short-term memory where visual memory implicated also auditory regions and, vice versa, auditory short-term memory was associated with the activity of the dorsal and ventral visual pathways (Michalka et al., 2015). Moreover, a recent work has shown fast modifications of functional connectomes and remarked the importance of even minute topological changes for the global network capacity to integrate information (Fransson et al., 2018). Eventually, our previous study focused on the alternating dynamics of segregation and integration in a visual working memory task suggested that the interchange of segregation-integration required a quasi-continuous coherent activation of most of the recorded cortical regions resulting in a global complex network orchestration (Zippo et al., 2018). Therefore, the observed involvement of global network dynamics appeared coherent with these recent results.

Despite the present study analyzed data from two independent experiments, it is however limited to a small sample of just 30 participants from a distinct younger age (23 years old on average, with small variance). Thus, for generalization of results, further investigations are needed considering larger populations homogeneously distributed in age. In addition, both studies referred to a single type of visual short-term and working memory task, thus, for more robust conclusions, similar studies with different experimental conditions and modalities (e.g., auditory or motor short-term learning) should be performed. The BOLD signal, generated by the functional MRI scanner is still considered an indirect measure of neuronal metabolism, unclearly linked with the synaptic activity, therefore, the robustness of the proposed results needed to be investigated in different experimental setups with more direct measures of the neuronal activity (e.g., EEG/MEG).

However, notwithstanding these limitations, it would be a curious exception that other sensory and cognitive tasks involved in the individual survival and environmental adaptation, could perform different topological dynamics. This could be due to the implicit law of parsimonious evolutionary conservation of basic schemes for coherent cognitive abilities.

In conclusion, the results of the work highlight the effectiveness of a global topological strategy in the treatment and storage of a task (in this case) a temporary visual memory retention, which drives the functional topologies towards more information processing optimized configurations. Novel interpretations of whole brain functional networks could therefore be envisaged in investigations regarding the brain cognitive functions.

## Data Availability Statement

Publicly available datasets were analyzed in this study. These data can be found here: https://openneuro.org/datasets/ds000002, https://openneuro.org/datasets/ds000052, and https://openneuro.org/datasets/ds000107.

## Author Contributions

AZ designed the study, performed the analyses, and wrote the manuscript. IC, JL, VB, MV, and GB revised all analyses and the manuscript.

## Conflict of Interest

The authors declare that the research was conducted in the absence of any commercial or financial relationships that could be construed as a potential conflict of interest.

## Footnotes

1 https://opendatacommons.org/licenses/pddl/index.html

2 nitrc.org/projects/artifact_detect/
